# Aging Worsens the Effects of Sleep Deprivation on Postural Control

**DOI:** 10.1371/journal.pone.0028731

**Published:** 2011-12-07

**Authors:** Rébecca Robillard, François Prince, Daniel Filipini, Julie Carrier

**Affiliations:** 1 Center for Advanced Research in Sleep Medicine, Hôpital du Sacré-Cœur de Montréal, Montréal, Québec, Canada; 2 Département de Kinésiologie, Université de Montréal, Montréal, Québec, Canada; 3 Institut Universitaire de Gériatrie de Montréal, Université de Montréal, Montréal, Québec, Canada; 4 Département de Psychologie, Université de Montréal, Montréal, Québec, Canada; University of Alberta, Canada

## Abstract

Falls increase with age and cause significant injuries in the elderly. This study aimed to determine whether age modulates the interactions between sleep deprivation and postural control and to evaluate how attention influences these interactions in the elderly. Fifteen young (24±2.7 y.o.) and 15 older adults (64±3.2 y.o.) stood still on a force plate after a night of sleep and after total sleep deprivation. Center of pressure range and velocity were measured with eyes open and with eyes closed while participants performed an interference task, a control task, and no cognitive task. Sleep deprivation increased the antero-posterior range of center of pressure in both age groups and center of pressure speed in older participants only. In elderly participants, the destabilizing effects of sleep deprivation were more pronounced with eyes closed. The interference task did not alter postural control beyond the destabilization induced by sleep loss in older subjects. It was concluded that sleep loss has greater destabilizing effects on postural control in older than in younger participants, and may therefore increase the risk of falls in the elderly.

## Introduction

The incidence of falls increases with age (Statistics Canada, 2002/2003). A recent epidemiologic study conducted in a sample of 666 individuals estimated that more than 60% of people between 50 and 90 years of age have a history of falling, mostly with multiple falls [1]. Importantly, in about 80% of cases, these falls reportedly caused injuries ranging from cuts and bruises to bone fractures and head injuries.

To maintain its balance, the body constantly produces reaction forces under the feet to counteract the movements of the center of mass [2]. The center of pressure (CoP) is the central application point for these reaction forces. Because the CoP constantly moves around the center of mass to maintain balance, dynamic parameters of the CoP displacement are commonly used to characterize postural control. Different configurations of these parameters reflect different postural states. For instance, wide, fast, and disorganized CoP displacements increase the likelihood of crossing postural stability boundaries, and are therefore commonly interpreted as reflecting a more unstable state. Accordingly, increased range, speed, and variability of CoP displacements during quiet standing have been associated with increased risk of falls [3], [4], [5]. On the other hand, slow and narrow CoP displacements with low variability produce an overly rigid or stiff postural control that is likely to reduce sensory feedback and the ability to adjust to perturbations.

Healthy aging leads to significant changes in postural control during quiet stance, including increased CoP amplitude and speed [5], [6]. Moreover, compared to non-fallers of the same age, elderly individuals with a history of falls use wider and faster movements to regulate their posture [7], [8].

Studies in young adults have shown that postural control is sensitive to sleep loss [9], [10], [11]. Even in healthy older adults with no specific sleep disorders, aging is accompanied by substantial changes in sleep quality and quantity. For instance, compared to younger participants, elderly participants have shorter sleep episodes and higher proportions of light sleep stages (stages 1 and 2) at the expense of deep sleep stages (stage 3 and 4, slow-wave sleep) [12]. Hence, poor sleep may contribute to age-related changes in postural control. However, the impacts of sleep deprivation on postural control in older adults remain poorly understood. Interestingly, an epidemiological study conducted in participants aged 66 years and older revealed that the occurrence of at least one fall in the last 12 months was associated with the presence of sleep disorders [13].

The impacts of sleep deprivation on postural control are likely to be modulated by environmental and internal factors such as sensory information and cognitive state. Vision allows online processing in order to adjust information about body movements and spatial orientation. In young adults, some studies suggested that sleep deprivation induces more body movement variance with eyes closed compared to eyes open [14], whereas we [11] and other authors [10] found no significant interactions between sleep and visual deprivation on postural variables associated with CoP displacement. Importantly, the degradation of the visual system with aging is associated with age-related changes in postural control mechanisms [15] and higher incidence of falls [16], [17]. Therefore, the impacts of sleep deprivation on postural control could be more pronounced in elderly people under altered visual conditions.

The influence of cognitive resources on postural control dynamics also changes with aging. When concurrently performing a cognitive and a postural task, young adults regulate their posture in a stiffer way [18], [19], [20], [21], [22], whereas older adults loosen their postural control [23], [24], [25], [26]. In addition, visual deprivation increases the impacts of a concurrent attention task on posture in elderly participants [27]. Furthermore, cognitive resources are known to modulate the effects of sleep deprivation on postural control in young participants [9], [11]. Therefore, the integrity of sensory and cognitive resources and their interaction could modulate the postural control reaction to sleep loss in elderly people.

We aimed to compare the effects of sleep deprivation on postural control and their modulation by attentional resources and visual input in young and older adults. We used the same experimental protocol we used previously to determine the effects of sleep deprivation on young adults in various postural conditions [11], and we used data from that study to compare the effects of age. Results revealed that sleep deprivation induces more perturbations in postural control following in older compared to younger adults, especially when the visual input is altered.

## Materials and Methods

### 1. Ethics Statement

Each participant signed a written informed consent form and received monetary compensation. This study was approved by ethical committee of the Centre de recherche de l'Hôpital du Sacré-Cœur de Montréal.

### 2. Participants

Fifteen healthy young adults (7 women and 8 men; 20–28 y.o., mean(SD) = 24(2.7)) and 15 older adults participated in this study (7 women and 8 men; 60–70 y.o., mean(SD) = 64(3.2)). All participants spoke French. Exclusion criteria were uncorrected visual impairment; use of medication known to influence sleep or postural control; sleep complaint or habitual sleep duration of less than 7 hours or more than 9 hours; history of auditory, postural, vestibular, psychiatric, or neurological disorder; night work or transmeridian travel three months prior to the study; and body mass index higher than 30. All participants scored lower than 10 on the Beck Depression Scale (long version; [28]). Blood sample analysis (complete blood count, serum chemistry including hepatic and renal functions, prolactine levels) and urinalysis results were checked by a physician for significant medical conditions. Prior to data acquisition, participants underwent a polysomnographic (PSG) adaptation and screening night with recordings from a nasal/oral thermistor and electromyogram (EMG) leg electrodes to screen for poor sleep efficiency, sleep apnea, and periodic leg movements. The presence of sleep disturbances such as sleep apnoeas and hypopnoeas (index per hour >10), periodic leg movements (index per hour >10), prolonged sleep latency (>30 min), or low sleep efficiency (<80%) resulted in exclusion from the study.

### 3. Procedure

Each participant was submitted to two counterbalanced experimental conditions separated by at least two weeks. One week prior to each condition, participants had to maintain regular self-selected sleep–wake schedules (±30 minutes for bedtime and wake time) and complete a French version of the Pittsburgh Sleep Diary [29]. Data from these diaries were used to calculate mean habitual bed and wake times in the laboratory and to schedule the experimental protocol (Mean habitual bedtime (SD), Young = 23∶41 (1∶05 hrs), Older: 22∶37 (0∶49 hrs); Mean habitual wake time (SD), Young: 7∶49 (1∶08 hrs), Older: 6∶55 (0∶34 hrs)).

In the sleep condition, participants slept in the laboratory according to their habitual sleep–wake schedule. In the sleep deprivation condition, participants were sleep deprived for 26 hours while a research assistant ensured that they did not fall asleep. For both conditions, participants performed the postural tasks two hours after their habitual wake time. In both sleep conditions, 0.5 to 2 hours prior to the postural tasks, subjects were provided with a light caffeine-free snack prepared by a dialectician to control for caloric intake.

#### 3.1 Postural tasks

To perform the postural tasks, participants stood upright on one or two adjacent AMTI force platforms (Advance Mechanical Technology Inc., Watertown, MA, USA) with feet at shoulder width. Foot placement was traced to ensure that the feet position was constant across trials. Postural tasks were conducted for 120-second periods under three cognitive loads: while performing an interference task, a control task, or while not performing any cognitive task. Participants were tested in all three cognitive loads with eyes open and eyes closed, for a total of six different postural conditions. Participants were asked to stand as still as possible and to look at a dot placed at about 1.1 meter in front of them (or an imaginary fixation point when they kept their eyes closed). In the dual task conditions (postural and interference or postural and control tasks), participants were instructed to divide their attention equally between the postural and cognitive tasks. All participants sat for resting periods of at least 30 seconds between each condition. The order of visual conditions was counterbalanced between participants: in each age group seven participants started the postural tasks with eyes open before they performed in eyes closed conditions, and eight participants started with eyes closed before they performed in eyes open conditions.

Details about the force platform data acquisition are reported elsewhere [11]. Two postural variables were extracted from the CoP time series in anterior-posterior (AP) and mediolateral (ML) directions: CoP Range (i.e. the distance between minimal and maximal CoP position) and CoP Speed (i.e. the mean of the instantaneous CoP velocities). These parameters were chosen because they provide information on the main components of postural control dynamics, i.e., the scale and velocity with which postural control operates. Moreover, we previously found these parameters to be sensitive to the effects of sleep loss and increased cognitive load [11].

#### 3.2 Auditory tasks

The interference task was designed and programmed using E-Prime (Psychology Software Tools, Inc., Pittsburg, PA, USA). Participants heard either identical or different French words (*high, low, soft,* and *loud*) in dichotic hearing through headphones. The words were pronounced in a congruent or incongruent voice according to their meaning (e.g., *high* was pronounced at low pitch or *soft* was pronounced at loud intensity). As quickly and accurately as possible, participants had to report verbally what they heard in the right ear while trying to ignore the stimuli introduced into the left ear. For the *high* and *low* stimuli, participants had to judge the voice pitch regardless of the word, and for the *soft* and *loud* stimuli, they had to state which word they heard regardless of voice intensity.

In the control task, the same four words were presented with no interference (i.e. between high and low pitch and between soft and loud intensity, and with the same stimuli in both ears), and participants had to produce the same verbal responses as in the interference task as quickly as possible. Detailed stimuli characteristics are reported elsewhere [11].

### 4. Statistical analyses

Two-way ANOVAs with an independent factor (2 age groups: young, older) and two repeated measures (2 sleep pressures: sleep and sleep deprivation) were performed on each postural variable measured in eyes open no task condition to assess the interactions between age and sleep pressure during the simple quiet stance. Each postural variable was also submitted to three-way ANOVAs with repeated measures (2 sleep pressures: sleep and sleep deprivation, 2 visual states: eyes open and eyes closed, and 3 cognitive loads: while performing an interference task, performing a control task, and not performing a cognitive task). In order to improve the normality of distribution, CoP Range AP and ML were log-transformed. P values for repeated measures with more than two levels were adjusted for sphericity with Huynh–Feldt corrections, but original degrees of freedom are reported. Post hoc Tukey HSD tests were used for multiple comparisons of means on significant main cognitive load effects and contrast analyses were used to decompose interactions. Statistical significance was set at a probability level of p<.05.

## Results

### 1. Comparison of the effects of sleep pressure on the young and older group

**Figure 1:**
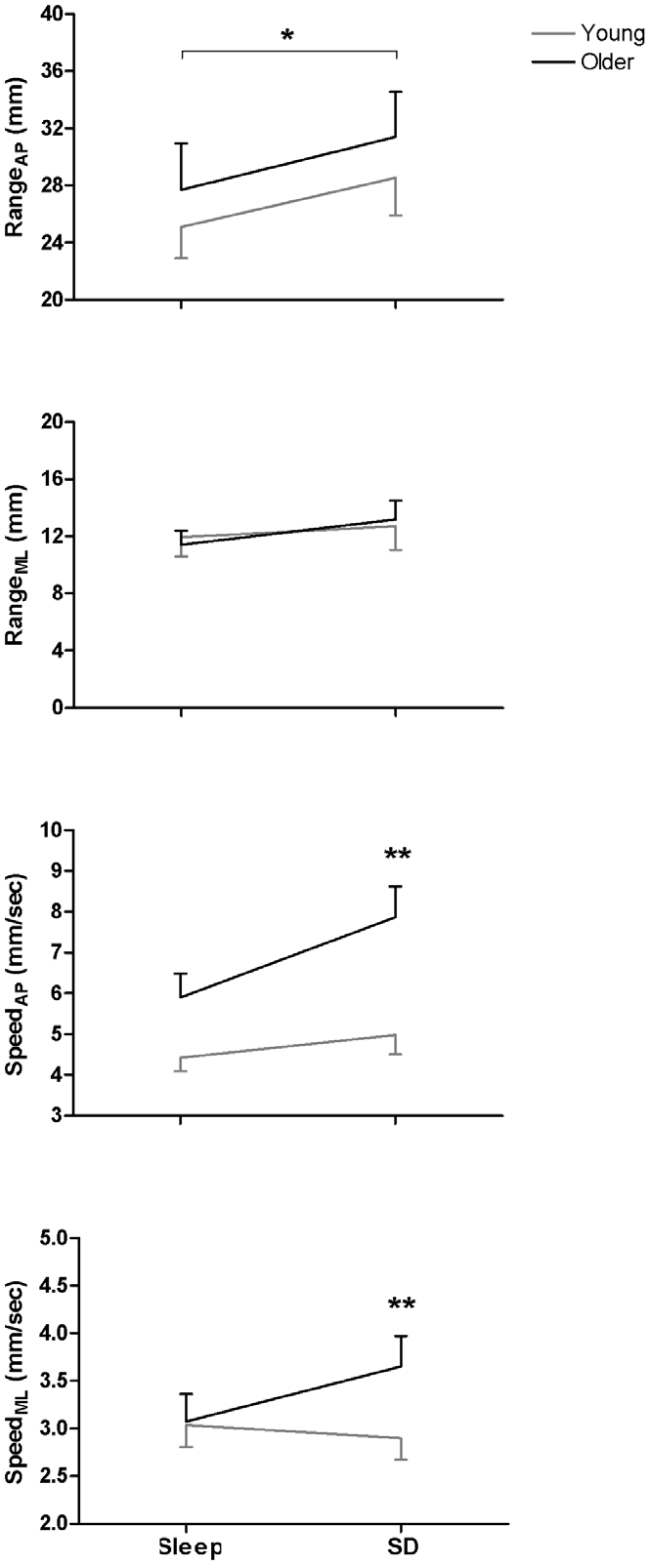
Age Modulation of the Effects of Sleep Loss on Postural Control. Means and SEM for each postural parameter after sleep (Sleep) and after sleep deprivation (SD) for the young group (n = 15; grey line) and the older group (n = 15; black line). * p<0.05, ** p<0.01.

Table 1 presents the two-way ANOVA results comparing postural variables between the two age groups and the two sleep pressure conditions in the baseline postural condition (i.e. eyes open and no cognitive task). Figure 1 shows the raw means and SEM for the four parameters after sleep and after sleep deprivation for each age group.

**Table 1:** Impacts of Age and Sleep on Postural Control Variables.

	Age	Sleep	Sleep by Age Interaction
	F(1,28); (p)	F(1,28); (p)	F(1,28); (p)
Range_AP_	0.4 (0.55)	**5.7 (0.02**)	0.1 (0.80)
Range_ML_	<0.1 (0.98)	3.8 (0.06)	0.6 (0.44)
Speed_AP_	**8.6 (<0.01)**	**23.9 (<0.01)**	**7.0 (0.01)**
Speed_ML_	1.2 (0.27)	2.6 (0.12)	**6.8 (0.02)**

Results of the two-way ANOVA (2 age groups by 2 sleep pressure conditions) with eyes open and no cognitive task. Significant effects and interactions are shown in bold.

#### 1.1 CoP Range

A significant main effect of sleep pressure revealed that CoP Range_AP_ was higher in the sleep deprivation than sleep condition (figure 1A). There was no significant main effect or interaction involving sleep pressure for CoP Range_ML_.

#### 1.2 CoP Speed

Significant interactions between age and sleep pressure were found for CoP Speed_AP_ (figure 1C) and Speed_ML_ (figure 1D). In both the AP and ML directions, contrast analyses showed that sleep deprivation increased CoP Speed relative to the sleep condition for older but not younger participants.

### 2. Effects of sleep pressure and postural conditions on older participants

**Figure 2:**
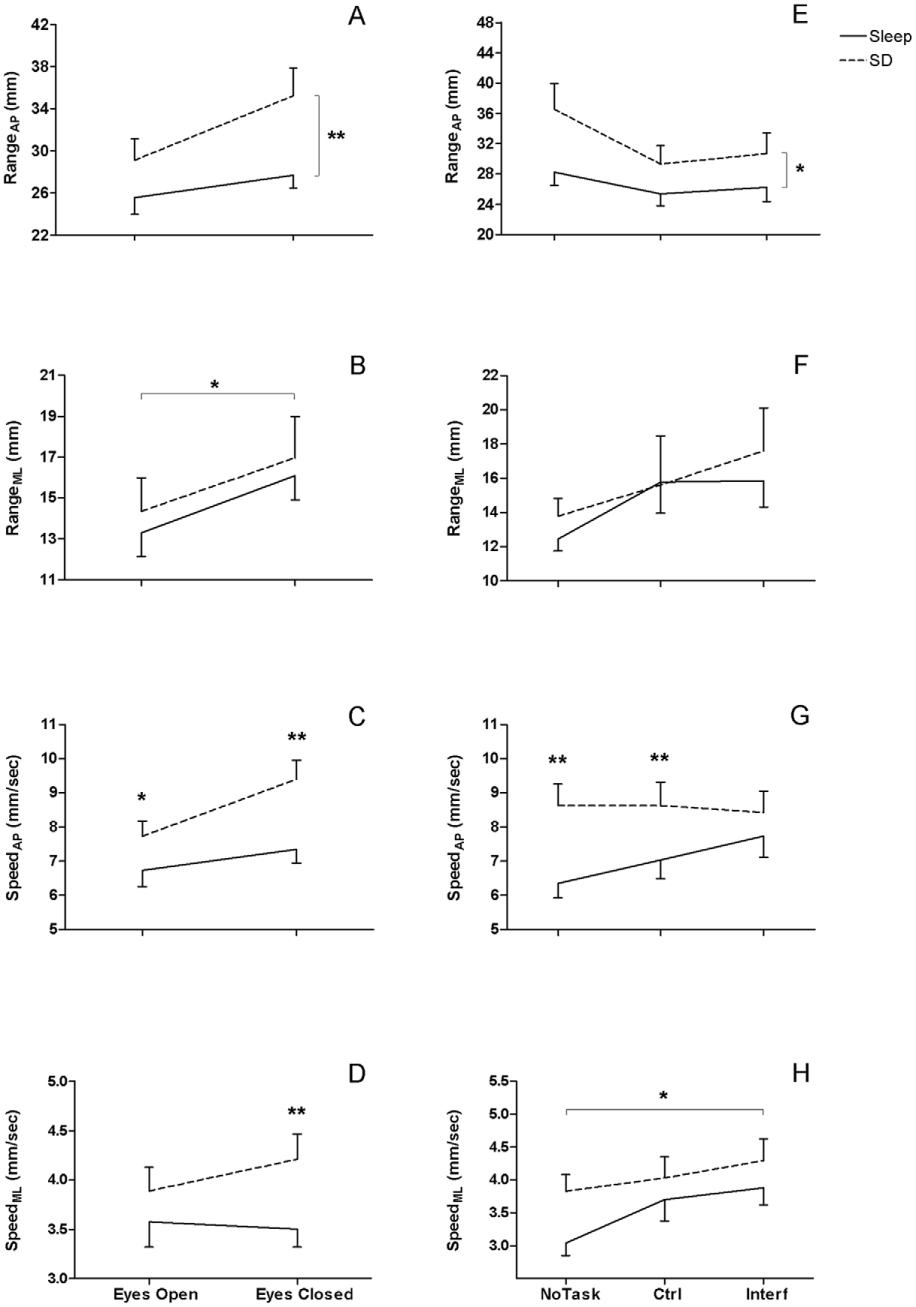
Interactions Between Sleep and Vision/Cognitive Load in the Older Group. Older participants' means and SEM for each postural parameter after sleep (black line) and after sleep deprivation (dotted line). Left panels (A to D): Effects of sleep deprivation in each visual state (with eyes open and eyes closed; * p<0.05, ** p<0.01). Right panels (E to H): Effects of sleep deprivation in each cognitive load (when not performing any task (NoTask), the control task (Ctrl) and the interference task condition (Interf); * p<0.05, ** p<0.01).

Table 2 presents the results from the three-way ANOVAs performed on the older group to compare postural variables in the two sleep pressure conditions, the two visual states, and the three cognitive loads. Figure 2 shows the significant effects and interactions revealed by these analyses.

**Table 2:** Impacts of Sleep, Visual State, and Cognitive Load on Postural Control Variables in the older group.

	Sleep F(1,14); (p)	Vis F(1,14); (p)	Sleep by Vis Interaction F(1,14); (p)	Cog F(2,28); (p)	Sleep by Cog Interaction F(2,28); (p)	Vis by Cog Interaction F(2,28); (p)	Sleep by Vis by Cog Interaction F(2,28); (p)
Range_AP_	**11.6 (<0.01)**	**13.0 (<0.01)**	1.3 (0.28)	**5.3 (0.02)**	3.0 (0.08)	**6.0 (<0.01)**	0.3 (0.72)
Range_ML_	0.01 (0.92)	**6.6 (0.02)**	1.0 (0.33)	2.2 (0.13)	1.3 (0.28)	1.7 (0.20)	1.4 (0.26)
Speed_AP_	**18.1 (<0.01)**	**37.0 (<0.01)**	**8.6 (0.01)**	1.6 (0.23)	**6.7 (<0.01)**	**3.8 (0.04)**	0.6 (0.55)
Speed_ML_	3.9 (0.07)	0.8 (0.40)	**5.2 (0.04)**	**4.8 (0.02)**	1.2 (0.31)	1.6 (0.21)	0.1 (0.89)

Results of the three-way ANOVA (2 sleep pressure conditions by 2 visual states by 3 cognitive load levels) performed in the older group. Vis: Visual state, Cog: Cognitive load. ns: non-significant effect or interaction. p-values were adjusted for sphericity with Huynh–Feldt corrections, but original degrees of freedom are reported. Significant effects and interactions are shown in bold.

#### 2.1 CoP Range

A main effect of sleep pressure indicated that CoP Range_AP_ was higher after sleep deprivation than after sleep (figure 2A and 2E). A visual state by cognitive load interaction indicated that CoP Range_AP_ was significantly higher in eyes closed compared to eyes open in the no task condition and in the control task condition, but not in the interference task condition.

There was no significant main effect or interaction involving the sleep pressure conditions for CoP Range_ML_. A visual state effect indicated that CoP Range_ML_ was significantly higher with eyes closed than with eyes open (figure 2B).

#### 2.2 CoP Speed

A significant sleep pressure by visual state interaction was found for CoP Speed_AP_ (figure 2C). Although CoP Speed_AP_ was higher in sleep deprivation than sleep condition for both visual states, this increase was higher with eyes closed than with eyes open. CoP Speed_AP_ also showed a significant sleep pressure by cognitive load level interaction (figure 2G). Contrast analyses showed that sleep deprivation increased CoP Speed_AP_ relative to the baseline sleep condition when participants did not perform any cognitive task and when they performed the control task, but not when they performed the interference task. A visual state by cognitive load interaction revealed that CoP Speed_AP_ was significantly higher with eyes closed than with eyes open in the no task condition and control task condition, but not in the interference task condition (figure 2C).

A sleep pressure by visual state interaction was also found for CoP Speed_ML_, showing that sleep deprivation significantly increased CoP Speed_ML_ over the sleep condition with eyes closed but not with eyes open (figure 2D). There was a main effect of cognitive load for CoP Speed_ML_, with significantly higher CoP Speed_ML_ for the interference task than the no task condition (figure 2H).

## Discussion

In this study, sleep deprivation had more destabilizing effects on postural control in older than younger adults. Furthermore, in the elderly, these effects were modulated by perceptual resources and the effects of high cognitive load did not seem to exacerbate the effects of sleep deprivation.

Our observations in conditions of unchallenged sensory and cognitive resources (i.e. in eyes open no task condition) revealed that postural control becomes more sensitive to sleep loss during senescence. Whereas the effects of sleep deprivation on postural control were restricted to the CoP range in the AP direction in young adults, they also affected CoP speed in both the AP and ML directions in the older group. Because CoP movements in the AP and ML directions are thought to be regulated by different postural control mechanisms [30], [31], this suggests that sleep deprivation may alter the biomechanics of postural control differently in young and older adults. The AP direction is regulated mainly by the ankle muscles, whereas postural control in the ML direction relies essentially on the hip abductor-adductor muscles. ML components of postural control are thought to gain importance in complex postural situations and to facilitate the initiation of a lateral step in order to restore balance [32], [33], [34]. We may postulate that age-related muscular atrophy heightens the ankle muscles' sensitivity to sleep loss and therefore increases the mobilization of the ML muscles during quiet stance under sleep deprivation. Moreover, because the hip muscles are more proximal to the center of mass than the ankle muscles, and therefore pose a lower inertial effect to counteract, older adults subjected to sleep loss could recruit the ML muscles to wield a more direct effect on the centre of mass.

Sleep deprivation increased the CoP range in young adults but increased both CoP range and speed in older adults. Therefore, in addition to shifting further from its central position, the CoP moved faster in sleep-deprived elderly participants, increasing the risk of crossing postural stability boundaries. Greater speed has previously been associated with higher fall rates in old age [4], [35], [36]. Hence, our results identify sleep loss as a potential risk factor for falls in the elderly, which is consistent with the frequent co-occurrence of falls and sleep difficulties independent of hypnotic use observed in epidemiological geriatric studies [37], [38], [39], [40]. Given the high prevalence of sleep difficulties in older people and the considerable consequences of falls in this population, the causal relationship between sleep loss and unstable posture calls for some overlap between interventions aiming to reduce falls and to reduce sleep problems in the elderly.

Because the postural control system can be influenced by multiple factors, identifying the conditions that modulate the impacts of sleep deprivation on postural control could provide empirical evidence for relevant prevention strategies. For instance, we found that altering visual input amplifies the increase in CoP speed that is induced by sleep deprivation. Because changes in postural variables following eye closure are thought to reflect the importance of the visual contribution to postural control, these results suggest that elderly people rely more heavily on visual information to stabilize their posture when they are sleep deprived than when well rested. Therefore, in older people, the risk of falling following sleep loss may be greater when the visual environment is dark or complex (e.g., a room with too much furniture, or an unfamiliar environment such as a hotel or hospital room), or when visual aids, such as glasses, are either inadequate or not worn.

In our older participants, the only postural variable that showed an interaction between sleep conditions and cognitive load was CoP speed_AP_, but this interaction is complex to interpret. The fact that the difference in CoP speed_AP_ after sleep and after sleep deprivation was significant only in the no task and control task conditions and not in the interference task condition suggests that increased cognitive load reduces the effects of sleep loss on postural control. However, as can be seen in Figure 2, CoP speedAP increased progressively with cognitive load in the sleep condition but remained relatively stable across the three cognitive loads in the sleep deprivation condition. The lack of significant difference between the two sleep conditions may be explained by a ceiling effect in the sleep deprivation condition. Therefore, our results suggest that high cognitive load and sleep deprivation are two factors that increase CoP speed in older adults, but that their effects are not cumulative.

The current study has some limitations that need to be considered. Laboratory technicians were instructed not to discuss any of the research hypotheses with the participants before they completed the study. Nevertheless, some participants may have foreseen some of the hypotheses, which could have influenced their performance across the sleep conditions. Moreover, even though the technicians were asked to provide standardized instructions for the postural tasks to participants in both sleep conditions, the technicians were not blind to sleep conditions. It is also important to note that some of the functional impacts of total sleep deprivation can differ to that of partial sleep deprivation (e.g. [41]). While the current study was limited to total sleep deprivation, future studies should evaluate if repeated partial sleep deprivation and sleep fragmentation have similar effects on postural control.

Our data suggest that the effects of sleep deprivation on postural control are more pervasive in older than young adults, making the regulation of upright posture more unstable and hazardous in the elderly. Importantly, postural instability in sleep-deprived elderly people worsened in poor visual conditions. Taken together, these results suggest that sleep loss is a significant risk factor for falling, especially in the elderly. To further understand the effects of sleep loss on postural control, future studies should manipulate the sleep pressure using sleep restriction and sleep fragmentation.
